# Intestinal Permeability, Inflammation and the Role of Nutrients

**DOI:** 10.3390/nu12041185

**Published:** 2020-04-23

**Authors:** Ricard Farré, Marcello Fiorani, Saeed Abdu Rahiman, Gianluca Matteoli

**Affiliations:** 1 Translational Research Center for Gastrointestinal Disorders (TARGID) Department of Chronic Diseases, Metabolism and Ageing (CHROMETA), KU Leuven, 3000 Leuven, Belgium; marcellofiorani94@gmail.com (M.F.); saeed.abdurahiman@kuleuven.be (S.A.R.); gianluca.matteoli@kuleuven.be (G.M.); 2 Centro de Investigación Biomédica en Red de Enfermedades Hepáticas y Digestivas (CIBERehd), Instituto de Salud Carlos III, 28029 Madrid, Spain

**Keywords:** mucosal barrier, epithelial integrity, nutrients, short-chain fatty acids, amino acids, vitamins

## Abstract

The interaction between host and external environment mainly occurs in the gastrointestinal tract, where the mucosal barrier has a critical role in many physiologic functions ranging from digestion, absorption, and metabolism. This barrier allows the passage and absorption of nutrients, but at the same time, it must regulate the contact between luminal antigens and the immune system, confining undesirable products to the lumen. Diet is an important regulator of the mucosal barrier, and the cross-talk among dietary factors, the immune system, and microbiota is crucial for the modulation of intestinal permeability and for the maintenance of gastrointestinal tract (GI) homeostasis. In the present review, we will discuss the role of a number of dietary nutrients that have been proposed as regulators of inflammation and epithelial barrier function. We will also consider the metabolic function of the microbiota, which is capable of elaborating the diverse nutrients and synthesizing products of great interest. Better knowledge of the influence of dietary nutrients on inflammation and barrier function can be important for the future development of new therapeutic approaches for patients with mucosal barrier dysfunction, a critical factor in the pathogenesis of many GI and non-GI diseases.

## 1. Introduction

The gastrointestinal tract (GI) allows physiologic responses such as nutrient and water absorption, water secretion on the one hand, and keeps luminal microbiota, noxious compounds, and undesirable products confined in the lumen on the other hand. A tightly regulated GI motility working together with annex glands such as the liver and pancreas can assure proper function of all these responses. The stomach receives and stores food for several hours and secretes acid and enzymes to favor proper digestion. During this period, the smooth muscle of the stomach contracts and relaxes to mix and break down the food to smaller particles that will be further processed in the duodenum. In the duodenum, the first and shortest segment of the intestine, digestion continues to prepare the chyme for the absorption of nutrients by adding bile from the gallbladder and digestive juices from the pancreas. The jejunum absorbs water and important nutrients such as amino acids, sugars, and fatty acids. The ileum, the most distal portion of the intestine, absorbs digestion products (for example vitamin B12) that were not absorbed before. The ileum is an important segment of the immune system of the GI tract due to the presence of Payer’s patches. Colon, the last part of the digestive system, reabsorbs the remaining water and prepares the luminal content for elimination. 

The so-called mucosal or intestinal barrier preserves the control and coordination of all these physiologic processes, which are in charge of maintaining normal homeostasis. The intestinal barrier not only protects the deeper layers of the intestinal wall, but also tightly regulates the passage of pro-inflammatory molecules, microorganisms, toxins, and antigens. Moreover, the mucosal barrier is continuously exposed to nutrients and other products present in the diet. In this review, we will describe (1) the different components of the mucosal barrier; and (2) how nutrients can modify different components of this barrier in physiologic conditions but also in organic (inflammatory diseases) and in functional pathologic conditions (functional GI disorders).

## 2. Structure and Physiologic Functions of the Intestinal Tract

**Figure 1:**
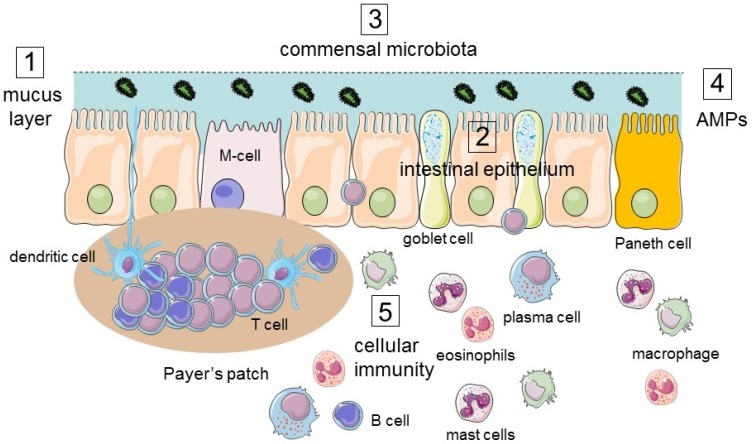
Summary of the different components of the mucosal barrier in the gastrointestinal tract (GI) tract. The physical elements include the (1) mucus layer, (2) intestinal epithelium, and (3) commensal microbiota. The immunological elements consist of (4) antimicrobial peptides secreted by Paneth cells and enterocytes, (5) cellular immunity. AMPs: antimicrobial peptides.

The different layers of the gut wall work in coordination to preserve GI homeostasis. The myenteric plexus (Auerbach’s plexus) controls the motility of the smooth muscle layers to propel the bolus along the gut while the submucosal plexus (Meissner’s plexus) plays a crucial role in controlling absorption and secretion of water. The coordination of both plexi favors a proper digestion of food and absorption of water and nutrients. The intestinal mucosa establishes an active semipermeable barrier which allows the absorption of nutrients and transport of substances and prevents the entry of harmful substances, luminal antigens, and pathogens. Distributed throughout the tissue layers, the mucosal barrier possesses different anatomical and functional elements (cellular and extracellular) to exclude these noxious substances (see Figure 1).

### 2.1. The Intestinal Mucosal Barrier

The intestinal mucosal barrier involves various types of cells and the complex interactions among them. Its complex function is based on the interaction of physical, biochemical, and immunological elements. The key component is the intestinal epithelium, which provides physical separation between the lumen and the internal milieu. 

#### 2.1.1. Lumen 

The gastrointestinal lumen consists of heterogeneous environments reflecting the specific functions of its different segments. The median gastric pH values differ from pH 1.4 to pH 4.6. In the proximal part of the small intestine, the pH ranges between 5.9 and 6.3. In contrast, in the distal part, the pH is significantly higher, ranging between 7.4 and 7.8. pH values fluctuate along the colon from pH 5 and pH 8 [1]. Interestingly, the gastric juice produced in the stomach has antibacterial properties not only due to its acidity, but also due to the presence of other components such as trypsin. Bile acids produced by the liver, released in the proximal duodenum, aid in the digestion of lipids. Its composition can affect intestinal permeability and has bactericidal properties. In the ileum, conjugated bile acids interact with enterocytes and promote the synthesis and secretion of antimicrobial factors from the epithelium [2]. 

Furthermore, the gut lumen contains different habitats that may contribute to the spatial heterogeneity of the microbiota [3]. Variation in microbial localization and density within the digestive tract has been described both longitudinally, from mouth to rectum as well as cross-sectionally, from the epithelium towards the lumen. The potential factors that can drive this heterogeneity are the above-mentioned chemical gradients (e.g., pH gradient, bile concentrations), radial oxygen distribution and nutrient availability, and immune interactions [4]. Bacterial colonization of the upper part of the gut is difficult and its bacterial density is estimated to be around 10^1^ to 10^3^ bacteria per gram of the contents in the stomach and duodenum. It increases to 10^4^ to 10^7^ bacteria per gram of content in the jejunum and ileum. The colon has the highest density, achieving 10^11^ to 10^12^ bacteria per gram, which contributes to 60% of total fecal mass [5]. Furthermore, beside microbial density, its ecology also diverges between the different segments of the GI tract. Biopsy samples from the stomach show an enrichment of *Lactobacillis*, *Veillonella,* and *Helicobacter*; samples from the small intestine have more *Firmicutes* and *Actinobacteria;* and colonic biopsies contain more *Bacteroidetes* and *Firmicutes* (especially from the *Lachnospiraceae* family) [5].

#### 2.1.2. Mucus Layer 

The mucus layer separates luminal contents from the epithelial compartment of the intestine. The mucus consists of water and glycosylated proteins called mucins secreted by goblet cells. The mucus prevents microbiota and large molecules from contacting the epithelial cells, but at the same time, allows passage of small molecules. The mucus layer also facilitates passage of the luminal contents longitudinally along the intestines and protects the epithelium from acid, digestive enzymes, and from microorganisms getting in touch with the epithelial layer. The mucus layer in the colon is composed of an inner and an outer layer. Intestinal microbiota is confined to the most external layer. In contrast, in the small intestine, the mucous layer is diffused and does not form a double layer [6]. 

Commensal microbiota can influence barrier function either directly by stimulating epithelial cell proliferation or by inducing the secretion of cytokines by epithelial cells and indirectly by synthesizing essential nutrients, vitamins, and short-chain fatty acids, which are an energy source for epithelial cells in the colon. Another important role of the microbiota is to shape the intestinal immune responses as well as priming the systemic innate immunity. The last important function of the microbiota is to inhibit colonization by pathogenic bacteria [7].

#### 2.1.3. Intestinal Epithelium

The intestinal epithelium is the key component of the intestinal mucosal barrier. It consists of a lining layer of epithelial cells composed of different cell types. The enterocytes are the most abundant cell type, forming an effective barrier to protect the internal milieu. In addition to its protective function, enterocytes control the selective uptake (absorption) of beneficial ions, nutrients, and other substances from the lumen into the body. Between the enterocytes, there are goblet cells, which are responsible for the secretion of mucus (gel-forming mucins) and enteroendocrine cells that produce GI hormones, peptides, and neurotransmitters. Paneth cells are mainly located at the crypt and are responsible for producing anti-microbial compounds that are important in immunity and host defense [8]. Also, overlying the Peyer’s patches in the small intestine are M cells, which along with goblet cells, play an important role in maintaining intestinal tolerance [9] (Figure 1).

Tight Junctions and Other Cell-to-Cell Adhesion Structures in Enterocytes: Evidences of the role of tight junction proteins as an important barrier are based historically on observations from very different fields. In 1976, using transmission electron microscopy, the junctional complexes between two epithelial cells were described for the first time in the gall bladder epithelium of a guinea pig [10]. In the most apical region of the epithelium, authors observed an intercellular gap of around 90 Å, which was named occluding zonule (zonula occludens), followed by adhering zonule (zonula adherens) with a gap of 200 Å and by the desmosomes with a gap of approximately 240 Å. The tight junction structure is responsible for the cell-to-cell adhesion complex that polarizes the intestinal epithelium, allowing the selective regulation of the ion passage, creating a potential difference at either sides of the tissue. The other structures provide structural support, anchoring the different types of epithelial cells. Hemi-desmosomes present in the basolateral membrane anchor the epithelial cells to the lamina propria.

Routes of Transport in the Epithelium: Paracellular, Transcellular, Transporter-Mediated, and Endocytic Pathways: There are several pathways for luminal products to cross the intestinal epithelium depending on the size, hydrophobicity, and other chemical characteristics of the product.

Small hydrophilic and lipophilic compounds can use the transcellular route to cross the plasma membrane of the enterocytes. Ions, water, and larger hydrophilic compounds between 400 Da and 10–20 kDa can certainly pass between enterocytes using the paracellular route. The tight junction proteins precisely regulate this route. Different nutrients such as amino acids, vitamins, and sugars cross the enterocytes using epithelial transporters that require energy (active transport). Larger peptides, proteins, and bacteria (endogenous microbiota or invasive bacteria) or large bacterial products cross the intestinal epithelium via endocytic vesicles, transcytosis, and subsequent exocytosis. Alterations in the paracellular pathway as well as in the uptake of large peptides, food antigens, and entire bacteria are supposed to be very relevant in the pathogenesis of several pathologies including gastrointestinal diseases.

Intestinal bacterial translocation is defined traditionally as the movement of viable micro-organisms from the gut lumen toward the mesenteric lymph nodes, but also to extraintestinal sites such as the peritoneal cavity, spleen, and liver [11]. Translocation of both whole bacteria and bacterial products such as endotoxins even occur in physiologic conditions. Lipopolysaccharide (LPS) is the main molecule located on the surface of Gram-negative bacteria (10–20 KDa in non-aggregated form) and has been observed to be increased in the plasma of patients with GI and non-GI diseases [12,13,14]. In addition, LPS-binding protein, another marker of recent microbial translocation, has been shown to be increased in the plasma of some of these patients [13,14]. The increased intestinal paracellular permeability, assessed using molecules of between 200 Da and 4 KDa found in these patients, has been suggested to be critical for the translocation of whole bacteria and bacterial products across the gut wall. Nevertheless, based on their molecular weight, only some forms of LPS and the major intestinal microflora-derived chemotactic peptides N-Formyl-l-methionyl-l-leucyl-l-phenylalanine (MW 437 Da) can potentially cross the epithelium through the paracellular pathway [15,16]. LPS has the capability of easily forming aggregates and complex structures of more than 100 KDa. In this configuration, LPS cannot cross the epithelium via the paracellular pathway [17]. 

The paracellular pathway is limited to molecules up to 20 KDa. Thus, commensal and pathogenic bacteria or large particles cannot cross the epithelium using this pathway. Bacterial uptake mainly occurs via the Peyer’s patches M-cells, but to a lesser extent also takes place via the enterocytes [18]. In contrast to the enterocytes, the brush border of M-cells is poorly organized with short irregular microvilli. This variation in morphology allows the internalization of bacteria, viruses, and large molecules in the intestinal lumen, which is then transported to the underlying lymphoid tissue. Independent of the material, particle internalization by the M cells is followed by the transport of endocytic vesicles containing the particle to the endosomal compartment and then to the basolateral membrane of the M cells by exocytosis [19]. In enterocytes, the uptake of enteric bacteria depends on the phosphorylation of the myosin light chain (MLC). Remarkably, this mechanism of whole bacterial internalization is controlled by IFN-γ, a cytokine that is also involved in the increased paracellular permeability to molecules up to 10 KDa, but not to small molecules of 200 Da [16].

Further Insights on the Paracellular Pathway and Its Modulation: Early studies in rats with experimental hemoglobinuria showed that between kidney tubule cells, hemoglobin (17 KDa) is concentrated in the tubular lumen and only permeates to the level of the tight junction and does not reach the deeper junctional complexes. The penetration of the tracer appears to be effectively stopped by the tight junction, indicating that the flux of large molecules across the paracellular space is exclusively regulated by them [10]. Subsequent studies using heavy metals such as La^3+^ demonstrated that the paracellular transport was specifically associated with the tight junction [20]. The epithelial tight junction structure is a dynamic and permeable barrier that plays a crucial role in physiologic processes such as water absorption and secretion as was described initially more than 50 years ago [21]. Recent research on the regulation of paracellular permeability has found that at least two types of pores are regulated by tight junctions. The first one is a high capacity and charge selective pore, which is permeable to small ions and small uncharged molecules (also known as “pore” pathway). The second one is a much larger pore with low-capacity (also known as the “leak” pathway), which is permeable to large ions and molecules regardless of the charge. Mainly claudin (CLDN) proteins regulate the pore pathway and in contrast, occludin and zonula occludens (ZO) proteins regulate the leak pathway. The permeability of both pathways can be measured using different, but complementary methods with increasing complexity. The transepithelial electrical resistance (TEER or Rt) measures the net movement of all ions (cations and anions) through the epithelium. TEER reflects the contribution of (1) the paracellular resistance (Rpara) which is regulated by the tight junctions; (2) the transcellular resistance (Rtrans) which indicates the resistance to ions of the apical (Rapi); (3) the basolateral resistance (Rbas); and finally, 4) the sub-epithelial resistance (Rsub). Unfortunately, the methodology to discriminate between the different types of resistance is complex and only accessible to a few research groups. TEER is evaluated using the Ussing chamber technique. In this technique, a current is applied across the epithelium and the potential difference generated is measured to calculate the resistance of the epithelium to the electric current using Ohm’s law. In simple words, TEER is the resistance that the epithelium exerts against the passage of ions. By using this technique, it was established that Cl^-^ and Na^+^ are the two most common ions in physiologic conditions mainly driving this current. Importantly, an increased permeability of the pore and the leak pathway reduces TEER, showing that small ions do not discriminate between both pathways. The permeability of the leak pathway can be assessed by measuring the flux of large molecules across the epithelium such as mannitol, sucrose, EDTA, inulin, and dextran molecules or polyethylene glycols (PEG) of variable molecular weights (from 4 up to 20 KDa). 

Both pathways appear to be regulated by local pro-inflammatory and anti-inflammatory cytokines. Among them, interferon gamma (IFN-γ), TNF-α, and interleukin (IL) 13 have received considerable interest. TNF-α, a Th1 cytokine, induces the contraction of the actin-myosin ring located in the most apical part of the enterocyte by the phosphorylation of the MLC, leading to the redistribution of the tight junction protein occludin as well as ZO-1. Functionally, this is translated with a reduced TEER and an increased flux of large molecules [22,23,24]. In contrast, the Th2 cytokine IL-13 involves a MLC kinase-independent mechanism, increasing the ion conductance (reduction of the TEER) but not modifying the paracellular flux of large molecules. Interactions between the leak and the pore pathway have also been described. The increased permeability of the leak pathway induced by the contraction of the actin-myosin ring after incubation with IFN-γ and TNF-α provokes the synthesis of IL-13 in the enterocytes that specifically up-regulates CLDN-2 expression and increases the cation ion flux. 

Another example of the interaction of both pathways is Th1 cytokine IFN-γ opening up a paracellular route for the entry of luminal antigens of high molecular mass (10 KDa) without gross disruption of the epithelial barrier to small molecules (180 Da) despite a consistent drop in TEER. This interaction between both pathways is mediated by CLDN-1 and the phosphorylation of occludin [16].

## 3. Responses of the Intestinal Tract to Environmental Challenges of the Lumen

### 3.1. Immune Response

Situated between the lumen and the underlying host immune system, the epithelial barrier acts as the first line of defense and checks the unfavorable activation of the host immune system against commensal bacteria. Mucus secreted by goblet cells and antimicrobial peptides (AMP) secreted by Paneth cells prevent the microbes from attaching to the epithelial cell boundary. AMPs are a diverse collection of ribosomally synthesized peptides with bactericidal properties against both gram-positive and gram-negative bacteria, mostly by disrupting their membrane integrity. In addition to defensins, Paneth cells also secrete in the intestinal lumen abundant quantities of other antimicrobial molecules such as lysozyme and phospholipase A2 (7).

Secretory immunoglobulin A (sIgA) produced by plasma cells in the *lamina propria* are transported by epithelial cells and secreted into the lumen. sIgA blocks epithelial-specific receptors on pathogens to prevent their attachment to epithelial cells [25]. Cellular immunity, also called immune barrier, comprises a variety of immune cells that reside in the *lamina propria* and in Peyer’s patches, both of which underlie the intestinal epithelium [26].

### 3.2. Structural Modifications of the Epithelium in Response to Luminal Challenges

#### 3.2.1. Innate Immunity

Innate immunity is the first line of immunological defense against pathogens present in the gut lumen and, in contrast to adaptive immunity, it is characterized by a lack of immunological memory. The main cellular constituents of this immune response are enterocytes, Paneth cells goblet cells, as well as dendritic cells, macrophages, neutrophils, mast cells, and eosinophils. This response is triggered by pathogen-recognition receptors (PRRs), which sense pathogen-associated molecular patterns (PAMPs) as well as damage-associated molecular patterns (DAMPs) that are generated by cells in response to different types of injury. Intestinal epithelial cells possess PRRs such as Toll like receptors (TLRs). TLR signaling in Paneth cells have been shown to elevate the release of AMPs in response to stimuli such as bacteria or bacterial products [27,28]. Basolateral TLR activation in the enterocytes elicits an inflammatory response characterized by the activation of the NF-κβ pathway and subsequent chemokine and cytokine production which is followed by the recruitment and activation of immune cells. Its role is essential to limit pathogen load and also to maintain the intestinal barrier function [25]. In response to luminal pathogens, mucosal macrophages, eosinophils and mast cells release toxic and inflammatory mediators such as histamine, nitrogen radicals, and Tumour Necrosis Factor alpha (TNF-α). Recruited neutrophils phagocytose and kill microorganisms by release of defensins and toxic enzymes such as lysozyme and peroxidase. However, this response is not always sufficient to avoid antigen penetration. Therefore, an immune adaptive immune response is then activated. 

#### 3.2.2. Adaptive Immunity

Luminal antigen exposure stimulates the adaptive immunity that is characterized by immunological memory (long-term protection) and that is crucial for the acquisition of oral tolerance. Adaptive responses are mediated by different immune cell populations such as macrophages, dendritic cells, mast cells, and lymphocytes including mechanisms such as antigen processing and presentation and production of regulatory and effector molecules. These molecules include cytokines, chemokines, and immunoglobulins. Furthermore, cytotoxic processes are activated against antigen invasion. 

Lymphocytes found lying between the epithelial cells are called intraepithelial lymphocytes (IELs). Intraepithelial lymphocytes (IEL) represent a unique cell population as they contain cell subsets expressing the αβ/γδ T-cell receptor chains and the αβ/αα CD8 co-receptor. IELs contribute to the barrier integrity and regulatory functions, but occasionally may become pro-inflammatory. They are predominantly T cells and are involved in the defense against viruses. They maintain epithelial integrity by promoting re-growth of healthy intestinal epithelial cells following cytolysis of epithelial cells during viral infection. They release interferon gamma (IFN-γ) and TNF-α as a response to an infection, which stimulate inflammation and intestinal barrier dysfunction [29,30]. 

The vast majority of large luminal antigens are mainly transported across the epithelium through M cells and are delivered to dendritic cells. Moreover, both dendritic cells and Cx3cr1^+^ macrophages have been shown to capture luminal antigens by transepithelial extension. Dendritic cells may also receive antigens from goblet cells and Cx3cr1^+^ macrophages and migrate to the draining mesenteric lymph node to activate T cells, resulting in a tolerogenic or immunoreactive response [31,32]. Following activation, naïve CD4 T lymphocytes may differentiate to different subsets based on the cytokine milieu: Th1, Th2, Th17, Th9, Th22, regulatory T (Treg) cells, and follicular helper T cells [33]. T lymphocytes including regulatory Foxp3+ T lymphocytes are found throughout the *lamina propria*, but especially in large numbers in Peyer’s patches. A high proportion of regulatory T cells in the intestine compared to any other part of the body are crucial for oral tolerance. The functions of various subtypes of T cells in the intestine are determined by their origin and development as well as by their interaction with antigen presenting cells in the *lamina propria* or in the gut-associated lymphoid tissue [34]. 

### 3.3. Maintenance of Homeostasis and Physiologic Functions

#### 3.3.1. Absorption of Glucose and Fructose

At low luminal concentrations, glucose absorption is transcellular, involving the apical Na^+^-glucose co-transporter SGLT1 and the basolateral Na^+^-independent, glucose transporter GLUT2, which transports glucose according to the concentration gradient. Absorption of glucose is coupled to an electrochemical gradient provided by the activity of Na^+^/K^+^-ATPase located at the basolateral membrane [35]. Nevertheless, after a meal, the luminal glucose can range between 50–300 mM, and about 30% of glucose absorption occurs through the leak pathway. The first evidences of this surfaced from studies using transmission electron microscopy, in which the apical surface of the enterocyte was exposed to glucose, then a condensation of the cytoskeleton was visualized indicating a contraction of the apical actomyosin ring [36]. The opening of the tight junction is initiated by transcellular glucose absorption by the SGLT1 [37], which then is followed by the phosphorylation of the MLC. The osmotic gradient created by transcellular Na^+^ and glucose transport enhances the paracellular flux of water, which in combination with the increased paracellular permeability, allows paracellular absorption of glucose and other nutrients dissolved [38]. However, given the fact that small intestinal glucose absorption is very fast, it is unclear whether sustained high luminal concentrations of glucose are sufficient to alter chronically intestinal permeability.

Consumption of commercial fructose has been increasing exponentially in the western diet, concomitantly with the increasing prevalence of obesity, metabolic syndrome, and nonalcoholic fatty liver disease. Several studies indicate that fructose may be a carbohydrate with greater obesogenic potential than other sugars [39]. It is widely accepted that fructose is absorbed through GLUT5 and GLUT2 transporters. Based on animal data, it has been suggested that consumption of high levels of fructose increases systemic endotoxin levels by increasing intestinal permeability, but results are not conclusive. Increased dietary fructose in mice for 6 weeks did not modify body weight but induced downregulation at the mRNA level of tight junction proteins of the leak pathway occludin and ZO-1 in the small intestinal. Nevertheless, the intestinal permeability to ions assessed by TEER measurements remained intact [40]. A longer fructose administration for 12 weeks showed similar results, with no changes in body weight but an increased permeability to PEG and dextran (4 KDa each), which was associated with the downregulation of occludin and ZO-1 at mRNA levels. Fructose administration induces higher levels of LPS in blood, increases the abundance of Firmicutes, and reduces the abundance of Bacteroidetes. Moreover, dietary fructose reduces mRNA expression of Muc2, α-defensin 1, and β-defensin 4, indicating an altered function of both goblet cells and Paneth cells, which are two other components of the mucosal barrier [41]. It is currently generally accepted that in physiologic conditions, the leak pathway can complement the transcellular pathway in absorbing nutrients but also water, especially when the membrane transporters are saturated [42]. 

#### 3.3.2. Secretion of Chloride Water

Secretion of water occurs in the entire GI tract from the duodenum to the distal colon in a similar proportion. It is very important to keep the epithelium hydrated to assure its correct function. The submucosal plexus tightly control secretion of Cl^-^ and water. Other mediators released by other intestinal cell subtypes can also modulate secretion (for instance, serotonin from enterochromaffin cells). The release of VIP and Ach by submucosal neurons stimulate the enterocyte, causing secretion of Cl^-^ as it is co-transported with sodium and potassium through the basolateral Na^+^-K^+^-Cl^-^ channel. Sodium is pumped back out via Na^+^-K^+^ ATPase while potassium is exported back via K^+^ channels. The activation of adenylyl cyclase by VIP leads to the generation of cAMP, which activates the cystic fibrosis transmembrane conductance regulator (CFTR), resulting in secretion of Cl^-^ into the lumen. Ach induces an increase in intracellular Ca^2+^ concentration, activating calcium-activated chloride channels and also inducing Cl^-^ secretion. Accumulation of Cl^-^ in the lumen creates an electric potential that causes Na^+^ to move across the tight junction into the lumen. The movement of Na^+^ ions into lumen creates an osmotic gradient, resulting in water being drawn into the lumen by an opening of the tight junction. The intake of several types of bacterial toxins like cholera toxin can permanently activate the adenylate cyclase in the enterocyte. This leads to elevated levels of cAMP, causing continuous opening of the CFTR channel. This results in a massive secretion of water, which is manifested as severe diarrhea [43]. 

#### 3.3.3. Absorption of Calcium 

The intestinal Ca^2+^ absorption is a crucial physiologic process for maintaining Ca^2+^ homeostasis in all organs and occurs through transcellular and paracellular pathways. In the proximal small intestine, the uptake of low luminal Ca^2+^ concentrations is transcellular. In contrast, in the distal small intestine, Ca^2+^ uptake is mainly paracellular as higher concentrations are present. The mechanisms involved in Ca^2+^ absorption via the enterocytes are poorly understood. Briefly, the transcellular route includes different steps: (1) entrance of Ca^2+^ into the enterocytes through the Ca^2+^ -selective transient receptor potential (TRP) channel TRPV6 and the voltage-gated calcium channel Cav1.3, a member of the L-type calcium channel family; (2) movement of Ca^2+^ to the basolateral membranes by binding to the high Ca^2+^ affinity protein calbindin-D 9 k; (3) basolateral extrusion of Ca^2+^ via plasma membrane Ca^2+^ ATPase PMCA1b; and 4) Ca^2+^ extrusion into the blood [44,45]. The paracellular pathway consists of Ca^2+^ transport through the pore-forming CLDN-2 and barrier-forming CLDN-12 tight junction proteins [46]. It remains to be further elucidated whether other proteins of the pore or the leak pathway are involved in Ca^2+^ absorption. Despite these two different mechanisms of Ca^2+^ uptake, there is evidence of crosstalk between the transcellular and paracellular pathways in intestinal Ca^2+^ absorption [47].

## 4. Beneficial Effects of Nutrients on Intestinal Integrity

### 4.1. Vitamins

Micronutrients such as vitamin A and vitamin D play key roles in the regulation of gastrointestinal homeostasis. Retinoic acid, a metabolite of vitamin A, mediates some of the effects. We have a nice compilation of pre-clinical and human studies showing that these fat-soluble vitamins are capable of affecting the different components of the mucosal barrier such as epithelial integrity, the innate and adaptive immune system, and gut microbiota. Both epithelial and a large number of immune cells in the GI tract express vitamin D and vitamin A receptors. These receptors are not expressed by Prokaryotes, suggesting that the described effects of these vitamins on the gut microbiota composition are probably indirect [48]. Vitamin A or D deficiency in animals reduces microbial diversity and increases the presence of commensals of Proteobacteria phylum [49] which are potentially pathogenic in patients with inflammatory bowel disease (IBD) [50]. Scarce evidences in humans showing that vitamin A-sufficient children have more diverse microbial communities compared with vitamin A-deficient children support these preclinical findings [51]. 

Mounting evidence indicates that vitamins also affect the intestinal epithelium. In vitro studies show that vitamin A and vitamin D are involved in the regulation of intestinal barrier function by modifying the expression of tight junction molecules. Intestinal epithelial cell lines treated with vitamin A or D have shown increased TEER, which is associated with the upregulation of ZO-1, occludin, and several claudins [52,53]. Deletion of VDR (VDR KO) in mice increases the susceptibility to DSS-induced colitis by inducing severe diarrhoea, rectal bleeding, and marked body weight loss, leading to death in 2 weeks when compared with normal animals. Moreover, colonic permeability to ions is increased as characterized by a reduced TEER [52] and the paracellular permeability to 4 KDa dextran is increased [49]. 

Accumulating evidences show that the regulation of innate and adaptive immunity mediated by vitamin A and vitamin D occurs at different levels of the mucosal barrier of the GI tract. It is remarkable that these vitamins are necessary for the correct development and function of type 3 innate lymphoid cells [54] and other types of immune cells of the gastrointestinal tract. The intestinal mucosa of vitamin A-deficient mice has fewer lymphocytes than normal [55]. Furthermore, vitamin D receptor knockout mice have shown fewer regulatory T cells expressing CD8αα in the gut. Interestingly, retinoic acid supplementation can increase the number of this population of T effector memory cells [56]. Vitamins are not only crucial for the development and maturation of some immune cell populations, but they also modulate immune responses at different levels. Retinoic acid is capable of increasing the expression of gut-homing receptors on the T cells such as α4β7 and the CC chemokine receptor 9 (CCR9) [57], while vitamin D reduces the homing process of T cells in the gut by decreasing α4β7 and CCR9 expression [58]. Both vitamins are capable of inhibiting IFN-γ production from T cells in vitro [59] and of inhibiting Th17 cells in vitro and in vivo. Retinoic acid and vitamin D can induce forkhead box P3 (FoxP3) protein and IL-10 production in vitro [60]. 

Retinoic acid can affect other components of the mucosal barrier—for instance, by stimulating the production of antibacterial peptides such as Reg3β and Reg3γ in enterocytes [61].

These and other findings suggest that the theoretically beneficial effects of vitamin A and D in humans may be due to the modulation of different components of the mucosal barrier. Nevertheless, only few studies or clinical trials are available in the literature. Interestingly, human studies have partially confirmed these previous findings in preclinical or in vitro models. In vitamin A-deficient children, vitamin supplementation improves the intestinal barrier function, assessed by the lactulose/mannitol test [62]. Moreover, short-term treatment with 2000  IU/day of vitamin D has been shown to improve small intestinal and colonic paracellular permeability and to reduce CRP plasma levels in patients with IBD [63]. 

As described above, it seems reasonable to postulate that vitamins may help reducing the increased intestinal permeability described in other pathologies like functional GI disorders, coeliac disease, and liver disease among others. However, this does not appear to have been investigated in published, controlled clinical studies.

### 4.2. Amino-Acids

In this section, we will focus on the effect of the amino acids glutamine and arginine. The effect of tryptophan on the mucosal barrier has been extensively reviewed recently (see review [64]). 

Absorption of amino acids is mediated by different types of transporters (active transport) localized in either apical or basolateral membranes of the intestinal epithelial cells in the small intestine [65]. Seven types of amino acid transporters are found at the apical surface of the enterocyte and at least five amino acid transporters are found at the basolateral membrane which can transport amino acids into interstitial fluid and then to the systemic circulation [66]. 

#### 4.2.1. Glutamine

Glutamine is a conditionally essential amino acid because its consumption increases during adverse conditions such as sepsis, trauma, and post-surgery recovery. Glutamine is the most important fuel for both enterocytes and immune cells. 

Deprivation of exogenous and endogenous glutamine in a Caco-2 monolayer cell model decreases TEER and increases the paracellular permeability to mannitol permeability. This functional alteration is associated with a downregulation and redistribution of the tight junction protein claudin-1, occludin, and ZO-1 [67,68]. Moreover, glutamine treatment in Caco-2 cells has a protective effect on acetaldehyde-induced epithelial barrier dysfunction. Glutamine ameliorates the acetaldehyde-induced reduction on TEER and the increased paracellular permeability to inulin. This improvement is associated with the restoration of the altered distribution of occludin and ZO-1 [69]. A recent study shows that pre-treatment with glutamine in vitro blocks the interleukin-13-induced intestinal epithelial barrier dysfunction characterized by a decrease of TEER and an increase of 4 KDa dextran permeability [70]. Glutamine deprivation not only affects the expression of tight junction proteins, but also reversely suppresses epithelial cell proliferation as shown in intestinal organoid cell culture [71]. Remarkably, glutamine deprivation does not affect the population of other cells such as Paneth and goblet cells, indicating that certain intestinal cell populations are more susceptible to specific amino acids deficiency. 

Extensive studies have shown the homeostatic regulation of cellular immunity by amino acids. It was found in an in vitro setup that glutamine reduces the production of pro-inflammatory IL-6 and IL-8 and enhances anti-inflammatory IL-10 level in T and B-lymphocytes and epithelial cells [72]. Also, glutamine can potentially modulate the innate and adaptive immune response since IL-10 plays an important role in the maintenance of intestinal mucosal homeostasis [73]. Depletion of glutamine blocked the proliferation of T cells and cytokine secretion [74]. In other words, glutamine is required for T cell activation. Activation of lymphocytes results in a metabolic reprogramming in which glutamine metabolism and its demand during inflammation are increased in specific cell populations. Indeed, an in vitro proton magnetic resonance spectroscopy study shows that glutamine levels in the colonic tissue of patients with IBD are decreased during active disease but not in patients in remission [75]. 

Several studies show that supplementation with glutamine attenuates intestinal inflammation and intestinal fibrosis in various preclinical models [76,77,78]. Nevertheless, research assessing the effect of glutamine on the epithelial function (intestinal permeability) in preclinical models is limited. Glutamine improved histological scores and inflammation and increased intestinal permeability of 4 KDa dextran in the small intestine of a preclinical model of ischemia-reperfusion (IR) injury and colitis [79,80,81].

In the past 30 years, many clinical studies of glutamine supplementation have been published for IBD treatment and other GI diseases (for example, see Table in [82]). Although the results of several of these studies seem convincing, they show several methodologic problems such as inadequate sample size, incomplete description of blinding, randomization procedures, and lack of a control group. Moreover, only few of them evaluate the effect of glutamine on the mucosal barrier. Despite the evidences of the effect of glutamine in preclinical models, there is currently insufficient evidence regarding efficacy and safety of glutamine for the treatment of Crohn’s disease, despite some trials showing positive results [83]. Interestingly, a randomized controlled trial showed that glutamine improves intestinal permeability assessed by the lactulose/mannitol test and morphology in patients with Crohn’s Disease. Nevertheless, similar results were found when whey protein was given in order to balance the control group in terms of the net protein intake [84]. In a recent study in patients with post-infectious diarrhoea-predominant IBS with increased intestinal permeability assessed by the lactulose/mannitol test, oral dietary glutamine supplements dramatically and safely reduced the symptoms and frequency of defecating and improved the consistency of stools and intestinal permeability [85]. 

Although many in vitro and in vivo data point to a beneficial role for glutamine in the maintenance of the mucosal barrier, larger, well-designed randomized trials are needed to better assess the beneficial effects of glutamine. 

#### 4.2.2. Arginine

Arginine is a semi-essential amino acid and a substrate for different enzymes such as arginases and nitric oxide synthases (NOS), among others. The constitutive form of NOS (cNOS) produces the small amounts of NO that are necessary for specific physiologic cell functions including maintenance of normal epithelial barrier in the intestine, as reviewed by Alican and Kubes [86]. Inhibition of cNOS by nitro-l-arginine methyl ester increases the paracellular permeability of the small intestine to smaller molecules but does not do so to larger molecules without inducing mucosal damage. It seems that this effect is mediated by an increased mast cell reactivity [87].

The inducible form of NOS (iNOS) is present in immune cells such as macrophages, neutrophils, and mast cells and is induced by the presence of inflammatory cytokines and endotoxins, resulting in the production of a high amount of nitric oxide. TLR agonist activation in macrophages can lead to NOS-mediated catabolism of arginine, which is a hallmark of pro-inflammatory (M1) macrophages while Arginase-mediated catabolism of arginine is implicated in suppressing the pro-inflammatory phenotype of macrophages [88]. 

In contrast to glutamine, only few studies show that arginine supplementation protects the intestinal epithelial integrity against different damaging conditions in different epithelial cell models. Pre-treatment with 4 mM of l-arginine partially reverts the drop on TEER and the increase in Lucifer Yellow (457.25 Da) paracellular permeability induced by heat stress conditions [89]. Arginine also prevents the reduction of TEER and increases in inulin paracellular permeability induced by hypoxia in jejunal IPEC-J2 monolayers. These functional changes are associated with a down-regulation of the tight junction protein ZO-1 [90].

Arginine can affect the maturation and functions of immune cells of *lamina propria*. The deficiency of arginine impairs B-cell maturation, leading to severely reduced B-cell numbers and number and size of Peyer’s patches. Moreover, it causes decreased serum IgM levels [91]. Since iNOS is present in macrophages, neutrophils, and mast cells, arginine deficiency affects innate and adaptive immunity. Many experimental and clinical data support the role of arginine and NO in immunity and inflammation, as has been reviewed by Wu et al. [92]. Oral supplementation of L-arginine improves intestinal immune function by increasing the amount of CD3+ and CD4+ T-lymphocytes and reducing the bacterial and endotoxin translocation in experimental acute pancreatitis in rats [93]. 

Alterations in arginine metabolism are present in animal models of colitis as well as in patients with IBD and arginine supplementation has been shown to ameliorate experimental colitis. Treated animals showed an improvement in body weight loss, a reduction in myeloperoxidase-positive neutrophils, and a reduction of the colonic paracellular permeability [94,95]. In other studies with pre-clinical models, dietary treatment with arginine shows amelioration of histological abnormalities and tight junction protein expression. However, in most of them, paracellular intestinal permeability was not assessed. L-arginine improves the jejunum morphology in a rodent model of heat stress-induced intestinal damage. This amelioration is associated with the upregulation of ZO-1 at mRNA and protein levels [96]. In another study, arginine supplementation mitigated the decreased tight junction protein expression of occludin and ZO-1 in the small intestine and promoted levels of bacterial endotoxin in plasma in a rodent model of non-alcoholic steatohepatitis [97]. Arginine supplementation reduced intestinal paracellular permeability of a molecule of 400 Da and bacterial translocation, assessed by gavaging with E.coli, in a model of intestinal obstruction [98]. Dietary arginine supplementation can also affect other compartments of the mucosal barrier. Arginine increased the abundance of bacteroidetes, reinforced the innate immune system by increasing the production of pro-inflammatory cytokines IL-1β, IFN-γ, and TNF-α, secretory immunoglobulin A, mucin-2 and mucin-4, and Paneth antimicrobials in the small intestine of mice [99].

Although there is a potential benefit for the use of arginine for the treatment of patients with an altered mucosal barrier based on the studies in epithelial cell models and pre-clinical models described above, to the best of our knowledge, no clinical trials have been reported. 

### 4.3. Fibers and Short-Chain Fatty Acids

Among the complex carbohydrates, dietary fibers (DF) are very relevant in the modulation of inflammation and gut barrier. The mammalian digestive system lacks the necessary enzymes to degrade DF, preventing digestion and absorption. Consequently, host’s microbiota ferment DF, resulting in the production of small organic metabolites, mainly short-chain fatty acids (SCFAs, such as acetate, propionate, and butyrate). There is a wide range of bacteria capable of fermenting DF, but according to enzymatic activity, each bacterium has a preferred substrate [100,101]. For the colonocytes, SCFAs are important energy substrates [102] and can also bind G-protein-coupled receptors (such as GPR41, GPR43, and GPR109a), acting as signaling molecules, regulating the immune responses and the differentiation of immune cells. Moreover, these molecules can induce an anti-inflammatory response. In fact, DSS-induced colitis is more severe in mice deficient in GPR43 and GPR109a [103,104]. In dendritic cells and colonic macrophages, GPR109a signaling promotes anti-inflammatory properties. Additionally, this receptor can induce the differentiation of Treg cells and IL-10-producing T cells [104]. Moreover, butyrate can enhance histone H3 acetylation in the promoter and other regions of the Foxp3 locus, increasing expression of Foxp3, resulting in lineage specification of Treg cells [105].

SCFAs are essential molecules involved not only in host metabolism and immunity, but also in intestinal barrier function. In fact, butyrate can improve the paracellular permeability of 3 KDa molecule regulating HIF1 (hypoxia-inducible factor-1), which modulates the efficiency of epithelial tight junction CLDN1 [106,107]. Moreover, studies, mainly using the in vitro Caco-2 cell monolayer model, have shown that butyrate enhances TEER without altering the expression, but affects the relocation of ZO-1 and occludin. We recently found that the treatment of primary intestinal epithelial cells of human colon organoids from patients with and without UC with butyrate increases TEER. Nevertheless, butyrate is detrimental for barrier function in the presence of inflammatory mediators TNF-α and IFN-γ [108]. Whether other tight junction proteins are implicated in the beneficial effects of butyrate needs to be further elucidated. Butyrate not only affects the epithelial function but also induces the goblet-cell-specific mucin MUC2 expression, the prominent component of the colonic mucus layer, in human goblet cell-like LS174T cells [109,110]. Consequently, a lack of dietary fibers and SCFAs production can compromise both epithelial barrier integrity and mucus production, altering gut permeability. 

Changes in DF intake can affect microbiota composition. In a mouse model, the lack of dietary fibers can impair intestinal barrier function, not only regarding reduced SCFAs production, but also because of some commensal bacteria, such as Akkermansia muciniphila. In the absence of DF, Akkermansia muciniphila utilizes host mucus glycans as a nutrient source, leading to alterations of the colonic mucus barrier and resulting in the development of colitis caused by the enteric pathogen Citrobacter rodentium [111].

## 5. Concluding Remarks

In addition to the obvious relevance of dietary nutrients in ensuring our survival and growth, they also play an important role in maintaining the homeostasis of different components of the mucosal barrier. Several studies have demonstrated that (1) nutrients play important roles in maintaining intestinal epithelium: growth of epithelial cells, homeostasis, and functions; (2) nutrients regulate the function of the intestinal epithelial barrier; (3) nutrients modulate the intestinal immunity; and remarkably, (4) nutrient supplementation has the potential to ameliorate the mucosal abnormalities present in patients with gastrointestinal disorders. 

This review provides further insight into the important roles of nutrients in homeostasis of the mucosal barrier and maintenance of the normal physiology of the intestine. More and well-designed clinical trials are needed to confirm the possibility of nutrient supplementation as a treatment for patients with mucosal barrier dysfunction, such as patients with IBD, coeliac disease, non-coeliac gluten sensitivity, irritable bowel syndrome, and functional dyspepsia.
